# Study of Postharvest Quality and Antioxidant Capacity of Freshly Cut Amaranth after Blue LED Light Treatment

**DOI:** 10.3390/plants10081614

**Published:** 2021-08-06

**Authors:** Siyuan Jin, Zhaoyang Ding, Jing Xie

**Affiliations:** 1 College of Food Science and Technology, Shanghai Ocean University, Shanghai 201306, China; krisjin190300741@163.com (S.J.); zyding@shou.edu.cn (Z.D.); 2 National Experimental Teaching Demonstration Center for Food Science and Engineering, Shanghai Ocean University, Shanghai 201306, China; 3 Shanghai Professional Technology Service Platform on Cold Chain Equipment Performance and Energy Saving Evaluation, Shanghai 201306, China

**Keywords:** freshly cut amaranth, light-emitting diode, antioxidation ability, microbial community

## Abstract

Freshly cut vegetables are susceptible to microbial contamination and oxidation during handling and storage. Hence, light-emitting diode technology can effectively inhibit microbial growth and improve antioxidant enzyme activity. In this paper, the freshly cut amaranth was treated with different intensities of blue light-emitting diode (LED_460nm_) over 12 days. Chlorophyll content, ascorbic acid content, antioxidant capacity, antioxidant enzymes activity, the changes in microbial count, and sensorial evaluation were measured to analyze the effects of LED treatment on the amaranth. Blue LED_460nm_ light irradiation improved the vital signs of the samples and extended the shelf life by 2–3 days. The AsA–GSH cycle was effectively activated with the irradiation of 30 μmol/(m^2^·s) blue LED_460nm_ light. According to the results, the LED_460nm_ light could retard the growth of colonies and the main spoilage bacteria, *Pseudomonas aeruginosa*, of freshly cut amaranth.

## 1. Introduction

Amaranth (*Amaranthus dubius* L.)is rich in ascorbic acid and other nutrients [1]. Freshly cut vegetables are ready-to-use products made from fresh vegetables after sorting, cleaning, and other treatment, which are convenient and fresh for consumers [2]. Cutting could cause mechanical damage to vegetables that would speed up their respiration rate. In addition, the cut wounds are susceptible to microbial invasion, which accelerates the deterioration of plant quality. There is a great demand for effective preservation techniques to maintain the quality of freshly cut commodities at this stage. Light energy is a necessary condition to maintain plant growth [3]. LEDs have characteristics such as low cost, high efficiency, and environmental protection. They have a wide range of applications in freshly cut vegetable preservation as well as in other areas with high research prospects [4]. Bhavya et al. [5] found that blue light could inhibit the proliferation of *Escherichia coli* and *Staphylococcus aureus*, and effectively improved the antioxidant enzyme activity of freshly cut pineapple slices. Zhai et al. [6] confirmed that UVC–LEDs could effectively sterilize *Escherichia coli* inoculated on freshly cut dragon fruit, while maintaining the quality of the freshly cut dragon fruit. Bian et al. [7] agreed that red and blue LEDs scavenged free radicals by enhancing the antioxidant capacity of lettuce while reducing nitrate levels. Maroga et al. [8] showed that 100 μmol/(m^2^·s) blue LED_450__nm_ light extended the shelf life of red freshly cut bell peppers, and improved antioxidant capacity and phenolic compounds. Chang et al. [9] confirmed that blue LED light irradiation enhances L-ascorbic acid content while reducing reactive oxygen species accumulation in Chinese cabbage seedlings. Samuolienė et al. [10] showed that blue LED irradiation of baby leaf lettuce had a significant positive effect on its DPPH scavenging capacity and enhanced its antioxidant properties.

In this paper, the physiological characteristics of freshly cut leafy vegetables treated with blue light were studied, and the bacterial colonies in the vegetables were assayed as well. It is short of research on antioxidant performance and antibacterial effects on freshly cut fruits and vegetables irradiated with monochromatic light (460 to around 470 nm). In this study, blue LED_460nm_ light was used to treat the freshly cut amaranth, and the changes in microbial total bacteria and antioxidant capacity, combined with physiological and biochemical indexes were measured to check the effects.

## 2. Materials and Methods

### 2.1. Treatment Method of Amaranth and LED Equipment Diagram

Amaranth plants were purchased from Shanghai Duoli farm fruit and vegetable cooperative. After amaranth plants were picked, they were quickly sent to the laboratory for treatment. Amaranth samples were cut about 5 cm away from the stem of the leaf, washed with ultrapure water, and dried naturally. Samples were chosen without yellowing or mechanical damage and freshly cut with a 1-cm scalpel. Samples were placed on a sterilized tray (80 g/plate), sealed with polyvinyl chloride (PVC) high-transmittance anti-fog film, and stored at 4 °C for 12 days.

**Figure 1:**
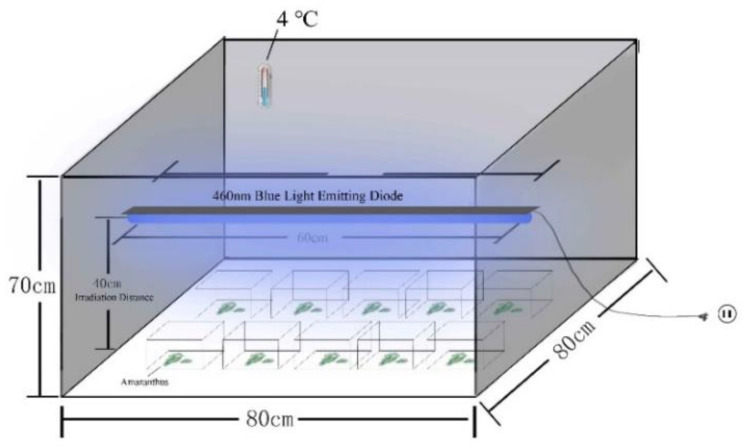
LED equipment diagram.

This experiment was divided into four groups, each group having 10 samples. The samples were irradiated with different intensities of blue LED_460nm_ light at 4 °C and relative humidity of 90 ± 5%. The irradiation height was about 30 cm. The irradiation time was 12 h per day. As shown in Figure 1, the T3 group had a 30 μmol/(m^2^·s) 460 nm LED light. Similarly, a 10 μmol/(m^2^·s) 460 nm LED lamp was installed in group T1, a 20 μmol/(m^2^·s) 460 nm LED lamp was installed in group T2, and no LED lamp was installed in group CK. The intensities were 0, 10, 20, 30 μmol/(m^2^·s), and related indicators were tested at two-day intervals for 12 days. The detailed treatments for each group are shown in Table 1. The physiological and biochemical indexes, total bacterial count, and antioxidant capacity of vegetables under the three light conditions were evaluated, where antioxidant capacity was assessed mainly by peroxidase, superoxide dismutase, and oxidative stress-related enzymes, and the corresponding data were processed.

### 2.2. Organoleptic Properties

Twelve consumers formed an evaluation group. The color, shape, and smell of the experimental samples were evaluated, reasonably and objectively. The full score of the three indicators was 10 points, and the specific scoring standards are shown in Table 2.

### 2.3. Soluble Solids

The soluble solids method referred to the method of Zhang et al. [11]. The samples were fully ground, and centrifuged at 4000 r/min for 10 min. The supernatant scale was added to the detection mirror to set the content of soluble solids, expressed by mass fraction (%), and repeated 3 times.

### 2.4. Weight-Loss Rate

The water weight-loss rate was calculated according to Formula (1).

(1) $$\mathrm{Weight}-\mathrm{loss}\;\mathrm{rate}/\%=\frac{M_{0}-M_{t}}{M_{0}}\times 100\%$$

### 2.5. Water Distribution and Migration

The water distribution and migration referred to the method of Bimal et al. [12].

### 2.6. Chlorophyll Content

Chlorophyll content was calculated according to the experimental method of Hasperué et al. [13]. The content of total chlorophyll was calculated according to Formula (2), the content of total chlorophyll, and the content of demethylated chlorophyll and carotenoid was calculated by:

(2) $$G/\%=\frac{\left(20.29*A_{645nm}+8.05*A_{663nm}*V_{T}*n\right)}{1000*m}$$

where *G*, the content of chlorophyll in a 1 g sample, mg/g; *A_645nm_*, the absorbance of the extract was measured at 645 nm; *A*_663*nm*_, the absorbance of the extract was measured at 663 nm; *V_T_*, total volume of extract, mL; *n*, dilution ratio of extract; *m*, fresh weight of freshly cut amaranth, g.

### 2.7. Ascorbic Acid Content

Ascorbic acid content referred to the method of Young et al. [14], with some modifications. A 1 g sample of amaranth leaf tissue was weighed and ground in 5 mL 0.05 mol/L oxalic acid. The supernatant was extracted by centrifugation at 4000 r/min for 10 min. All experiments were performed three times.

### 2.8. Ascorbate Peroxidase (APX) Activity

APX activity referred to the method of Zhao et al. [15].

### 2.9. Glutathione Reductase (GR) Activity

GR activity referred to the method of Giacomo et al. [16].

### 2.10. Peroxidase (POD) Activity

POD activity referred to the method of Zhao et al. [17].

### 2.11. Superoxide Dismutase (SOD) Activity

SOD activity was determined by monitoring the inhibition of photochemical reduction by nitroblue tetrazolium (NBT) [15].

### 2.12. Malondialdehyde (MDA) Content

MDA content refers to the method of Young et al. [18], with some modifications. A5 mL measure of 100 g/L trichloroacetic acid solution was added to a 1 g sample of amaranth. After homogenizing, samples were centrifuged at 10,000× *g* for 20 min at 4 °C. Next, 2 mL of supernatant was added to 2 mL of 0.67 g/100 mL thiobarbituric acid solution. After mixing, the solution was boiled for 20 min in a boiling water bath. The solution was centrifuged at 10,000× *g*, cooled with water. The experiment was repeated 3 times.

### 2.13. Aerobic Plate Count and Specific Spoilage Organism (SSO) Count

The aerobic plate-counting method referred to the method of Zhang et al. [11]. A total 10 g of freshly cut amaranth samples were weighed on the sterile operating platform, and homogenization solution with a ratio of 1:10 was prepared in the sterile bag according to the gradient dilution method. The homogenization solution was evenly placed on the plate-counting agar medium (PCA), and the colonies were tested by the inverted plate method. The specific spoilage organism(SSO)count referred to the method of Amal et al. [19].

Where *M_0_* is the quality of amaranth before storage; *M_t_* is the quality of amaranth during storage time *t*.

### 2.14. DNA Extraction and PCR Amplification

Microbial DNA was extracted using the HiPure Soil DNA Kits (Magen, Guangzhou, China) according to the manufacturer’s protocols. The 16S rDNA V5-V7 region of the ribosomal RNA gene was amplified by PCR using primers799F: AACMGGATTAGATACCCKG; 1193R: ACGTCATCCCCACCTTCC [20].

### 2.15. Illumina Novaseq 6000 Sequencing

Amplicons were extracted from 2% agarose gels and purified using the DNA Gel Extraction Kit according to the manufacturer’s instructions and quantified using ABI StepOnePlus Real-Time PCR System. Purified amplicons were pooled in equimolar and paired-end sequenced on an Illumina platform according to the standard protocols.

### 2.16. Data Analysis Method

Three parallel measurements were performed in all experiments. All data were expressed as average values ± standard error (*n* = 3); All data were performed by one-way analysis of variance (ANOVA) and the differences among the means were compared by Duncan’s multiple range test with a significance of *p* < 0.05 using the SPSS 17.0 statistical program (SPSS Inc., Chicago, IL, USA).

## 3. Results and Discussion

### 3.1. Changes in Organoleptic Properties and Shelf Life

**Figure 2:**
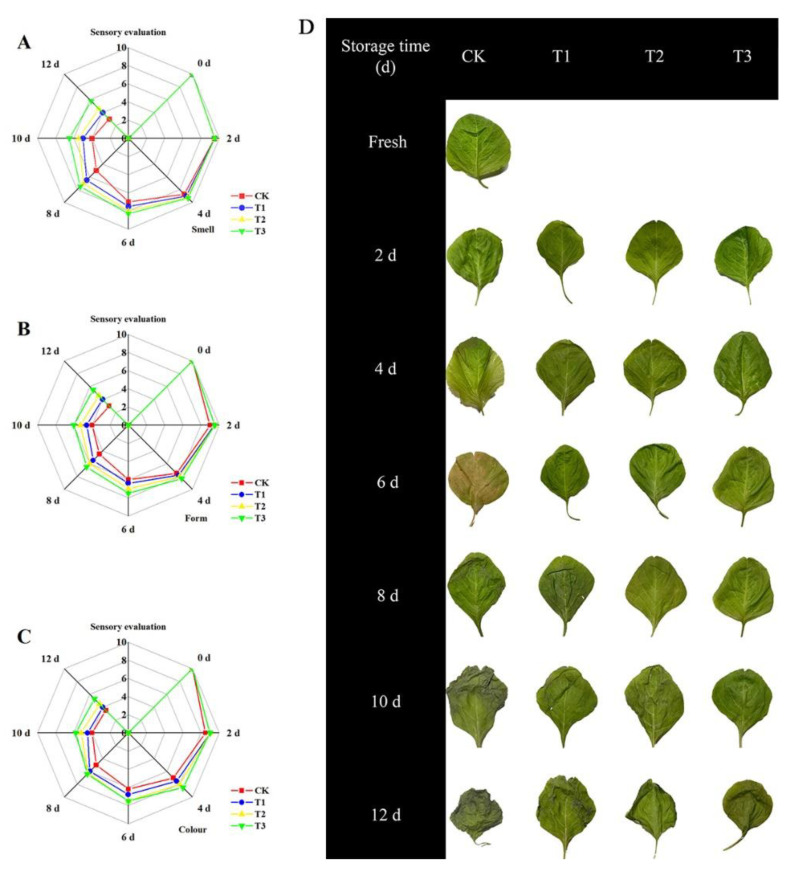
Changes in smell (**A**), form (**B**) and color (**C**) sensory characteristics, and sample shelf-life changes (**D**) during storage.

Sensory evaluation shows the freshness of freshly cut amaranth most intuitively and objectively [21]. In Figure 2, the color, appearance, and odor of freshly cut amaranth decreased during storage time. Significant differences were present in the irradiated and control groups. Onwards of the 8th day, the control group scored below 6 marks on each item. This indicated the deterioration of the quality of freshly cut amaranth in the control group. The leaves of each treatment group presented various degrees of water loss, appearing wrinkled and yellowed on the 10th day. The overall score was: T3 > T2 > T1 > CK. During storage, the sensory scores of the treatment groups were much higher than those of the control group. Blue LED_460nm_ light can slow down the aging of freshly cut amaranth and prolong the shelf life of freshly cut amaranth. Aiamlaor et al. [22] demonstrated that blue light irradiation was effective in delaying broccoli floret yellowing to extend the shelf life. Shelf-stage differences showed a close correlation with chlorophyll content. The chlorophyll content in the CK group appeared to decrease significantly at the 6th day. Yellowing was also evident at the 6th day during the shelf period. In all, the T3 treatment group had the best effects.

### 3.2. Changes in Soluble Solids Content

Sugars, vitamins, and minerals are the prime soluble solids in plants. Soluble solids can corroborate the consumption rate and maturity of plant nutrients. Certainly, an important indicator measures the freshly cut fruit and vegetable preservation effects [23,24].

**Figure 3:**
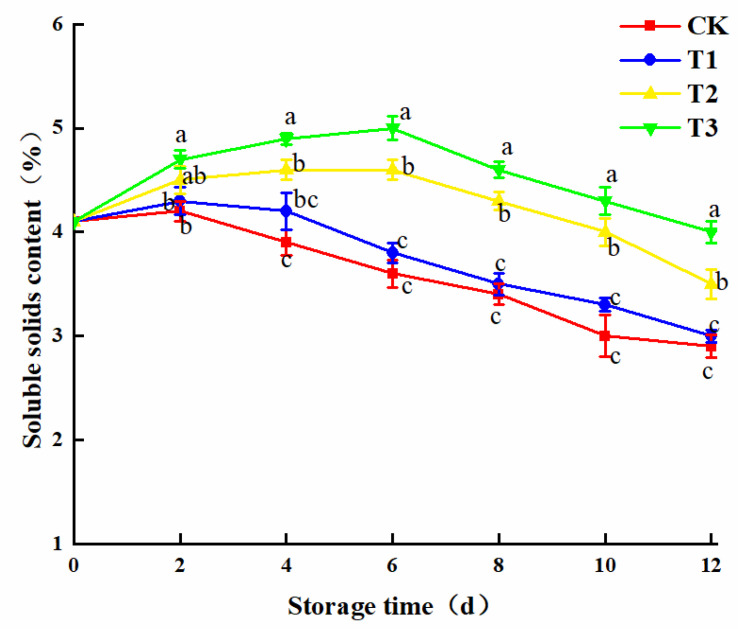
Changes in soluble solids content The means with different lowercase letters (a–c) in the figure differ significantly (*p* < 0.05).

In Figure 3, the soluble solids of the T2 and T3 treatment groups were able to remain in a stable range at the beginning of storage, which might be due to the enhanced respiration and vital activity of freshly cut amaranth, causing the balance between the consumption and formation rate of soluble solids. The plant vital signs decreased with storage time, resulting in increased consumption of soluble sugars and a decreased trend in the later stages of storage. Dramatic differences were observed between the T2 and T3 treatment groups and the control group (*p* < 0.05). It may happen that higher light intensity can better stimulate photosynthesis in amaranth and further synthesize more organic matter. No distinct differences were observed between the T1 treatment group and the control group, which might be due to insufficient light intensity to reach the light supplementation point of the freshly cut amaranth. The CK group was unable to photosynthesize under sheltered conditions in a more effective way. Noelia et al. [25] also confirmed the results.

### 3.3. Changes in Weight Loss and Moisture Migration

**Figure 4:**
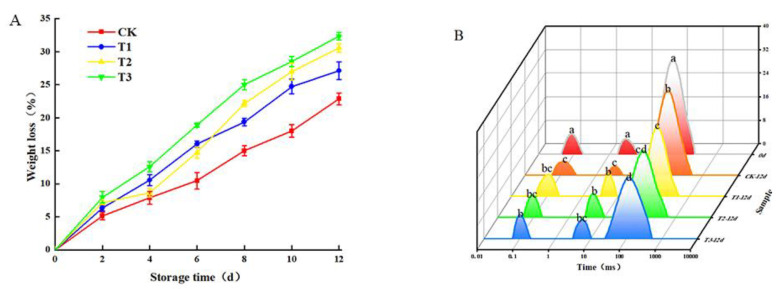
Changes in weight loss (**A**) and moisture migration (**B**). The means with different lowercase letters (a–c) in figure differ significantly (*p* < 0.05).

The loss rate of plant weight is mainly reflected in water loss and nutrient consumption. In Figure 4A, the weight-loss rate was: T3 > T2 > T1 > CK. This result indicated that blue light could effectively enlarge the stomata of plant leaves, and stomatal conductance increased with the increase of blue light intensity [26]. Sander et al. [27] also proved that blue light could trigger the qualitative signal effect of plants and photosynthesis again. Moreover, blue light could effectively accelerate the transpiration rate, further increasing the mass loss rate [28]. The samples were wrapped by PE film during the experiment, so the experimental results were controlled in a reasonable range.

In Figure 4B, the water migration of bound water (0–2 ms), immobilized water (2–20 ms), and free water (20–100 ms) in the leaves of the samples at the early stage of storage (0 d) and the end of storage (12 d) was obtained by inversion and calculation.

Water content was observed by peak value and peak area. In the first 12 d, the bound water and immobilized water content for the treatment groups showed an upward trend, at the same time as the content of free water fell. This may be due to the mechanical damage caused by self-healing of freshly cut amaranth and the synergistic effect of blue light irradiation to stimulate the stress response of the amaranth [29]. The increase in the bound water content showed that the stress resistance of the treated group was enhanced to a certain extent [3] The bound water content for the control group decreased at the end of storage, with significant differences among the other groups (*p* < 0.05). The possible reason behind the difference is due to the high metabolic reaction caused by light, which feeds freshly cut amaranth with energy in an efficient way. Combined with sensory evaluation index, the quality of freshly cut amaranth deteriorated significantly on the 12th day. This indicated that the metabolic system of freshly cut amaranth collapsed and could not maintain the plant body signs. At the end of storage, the free water content for each experimental group decreased. The consequence was: CK > T1 > T2 > T3. After blue light irradiation, freshly cut amaranth had vigorous life activity, metabolic activity, and oxidation reactions, which consume a lot of water. The results of moisture migration experiments corresponded to the weight-loss rate.

### 3.4. Changes in Chlorophyll Content

**Figure 5:**
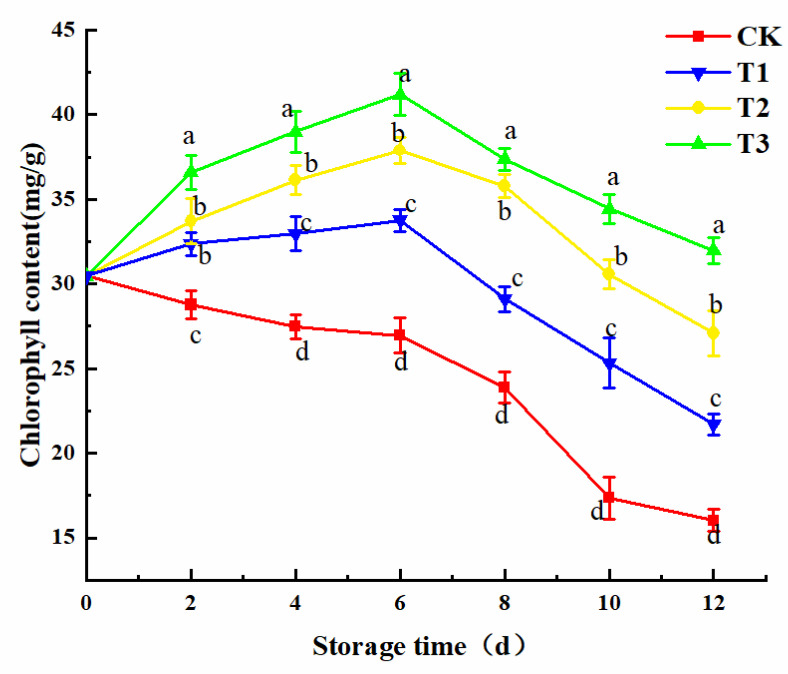
Changes in chlorophyll content. The means with different lowercase letters (a–d) in figure differ significantly (*p* < 0.05).

Chlorophyll is an important indicator of plant vital signs in the photosynthesis of plant cells. Chlorophyll can absorb light energy to synthesize carbohydrates, carbon dioxide, and water. Light can affect photosynthesis of plant cells by changing the absorption and consumption of light energy and electron transport [30]. In Figure 5, the chlorophyll content of the treatment groups irradiated by blue LED_460nm_ light show a trend of increasing from 0–6 days and then decreasing after the 6th day, with peak value occurring at day 6 (*p* < 0.05). The peak value of the T3 treatment group reached 41.18 mg/g. The chlorophyll of the control group decreased continuously with storage time. Bukhov et al. [31] found that barley leaf seedlings could synthesize chlorophyll more effectively under blue light irradiation; and the quantity of carotenoids, which could consume the energy of excessive excitation of chlorophyll and maintain the balance of physiological activities of the plants, increased under blue LED light. Light stimulated the activity of magnesium chelatase, then increased the content of chlorophyll. The results showed that the treatment of freshly cut amaranth with 30 μmol/(m^2^·s) blue LED_460nm_ light could effectively improve the chlorophyll content. The sensory score was consistent with the result, which could inhibit the quality deterioration of freshly cut amaranth for 2 or 3 days.

### 3.5. Changes in AsA Content and Oxidative Stressase Activity

**Figure 6:**
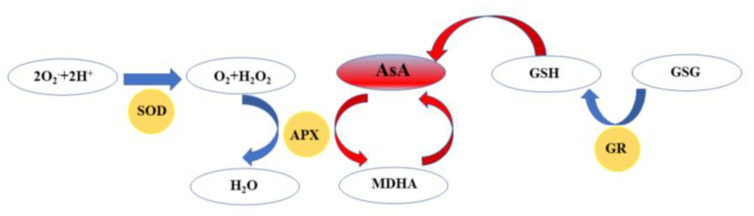
Mechanism diagram of AsA–GSH.

AsA can remove ROS and H_2_O_2_ indirectly through APX, because of the reducibility. AsA synthesis pathway in plants is called the Smirnoff Wheeler cycle. The oxidative stress process of AsA is shown in Figure 6. APX and GR were particularly important in the AsA-GSH cycle. APX could scavenge reactive oxygen species (ROS) and prevent oxidative damage in plants. Plant stress resistance could also be corroborated by the increase of APX activity. GR promoted AsA indirectly by regulating the dynamic balance of Glutathion (GSH), which was particularly important in the ROS removal process.

**Figure 7:**
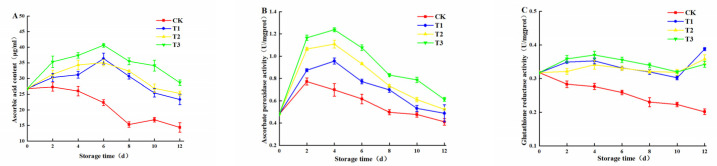
Effects of blue LED_460nm_ light with different intensities on AsA content (**A**); APX activity (**B**); and GR activity (**C**) of freshly cut amaranth.

As shown in Figure 7A, AsA content in the treatment group increased and decreased late, and reached the peak in the T3 experimental group on the 6th day in addition. The control group occurred a continuous decline. Ohashi et al. [32] confirmed that blue light can effectively improve the content of AsA in leafy vegetables. Combined with the APX activity and GR activity in Figure 7B,C, APX activity of all samples rose then decreased. In addition, the APX activity of the treatment group was significantly higher than that of the control group. GR activity in the treatment group remained at a stable level, while GR activity decreased for the control group. Blue LED_460nm_ light could effectively regulate the AsA–GSH cycle and increase AsA content in the end. Blue light was more effective in increasing AsA content compared to other light sources, as was demonstrated by Mishra et al. [33]. Rabelo et al. [34] confirmed that high light intensity could promote the production of photosynthetic products and further increase the accumulation of AsA. The activities of APX and GR were positively correlated with AsA content under different environmental stresses [35]. In conclusion, the freshly cut amaranth irradiated by blue light could increase ASA content, enhance the activity of GR and APX, and further excite the AsA–GSH cycle, especially 30 μmol/(m^2^·s) LED_460nm_ blue light had the best effect.

### 3.6. Changes in Antioxidant Enzyme Activity

**Figure 8:**
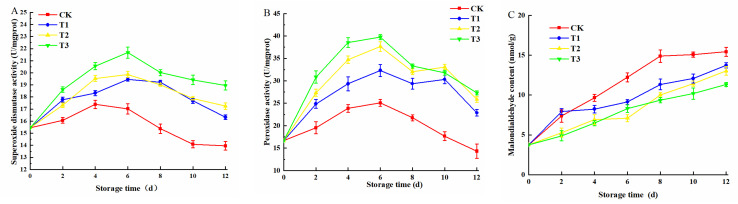
Effects of blue LED_460nm_ with different intensities on SOD activity (**A**); POD activity (**B**); and MDA contents (**C**) of freshly cut amaranth.

It can be seen from Figure 8A,B that the antioxidant enzymes of freshly cut amaranth irradiated by blue LED_460nm_ light ascended then descended. In the ascending phase, blue LED_460nm_ light induced an increase in POD and SOD activities. The activities of SOD and POD in the T3 treatment group reached the peak on the 6th day, increased by 27.33% and 58.49%, respectively, compared with the control group. With the increase of storage time, the photosynthesis and respiration activities in plants weakened, and the antioxidant enzymes activity began to decline. On the 8th day, the antioxidant enzymes of the control group were lower than the initial value. Combined with the sensory indexes, the control group had lower antioxidant enzymes than the original values on the 8th day. Figure 8 revealed that blue LED_460nm_ light can effectively enhance the activity of antioxidant enzymes. SOD mainly disproportionates O_2_^−^ and H^+^ to form O_2_ and H_2_O_2_. When the rate of O_2_^−^ scavenging by SOD was lower than the rate of O_2_^−^ production, as the dynamic equilibrium was broken, freshly cut amaranth accelerated its aging process [36]. POD and H_2_O_2_ were oxidized to produce phenolic free radicals to scavenge oxygen free radicals [37]. The accumulation of phenolic free radicals led to the increase of chlorophyll content and the degradation of chlorophyll in freshly cut amaranth. Blue light could effectively activate the antioxidant defense system of plants, such as *Stevia rebaudiana* [38], *lettuce* [39], and *carnation* [40]. The increase of antioxidant enzyme activity not only helped to remove ROS, but reduced the irreversible damage caused by photooxidation [41].

MDA content is an important signal of peroxidation of plant somatic cell membrane during storage. In Figure 8C, MDA content was decreasing. On the 6th day, the measurement was: T3 < T2 < T1 < CK. The results were consistent with the data of POD and SOD activities on the 6th day, indicating that the freshly cut amaranth treated with blue LED_460nm_ light could effectively reduce the degree of oxidative damage, minimize the damage of cell membrane and cell structure, and lessen the accumulation of MDA content. Li et al. [42] confirmed that *Chinese Kale* irradiated by blue LED_460nm_ light could effectively delay the accumulation of MDA.

### 3.7. SSO Count and Aerobic Plate Count

**Figure 9:**
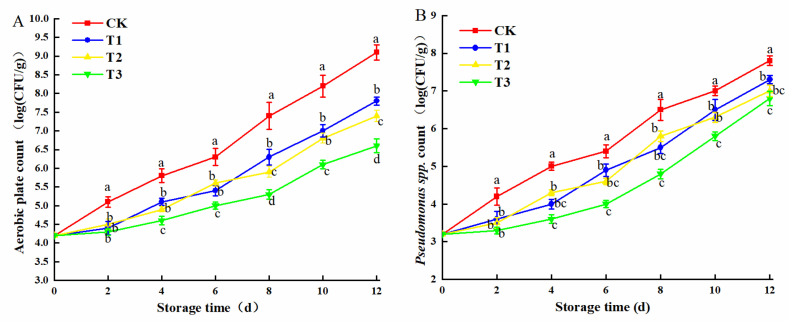
Changes in aerobic plate count (**A**) and *Pseudomonas spp.* count (**B**). The means with different lowercase letters (a–d) in figure differ significantly (*p* < 0.05).

In Figure 9A, the total number of colonies in the sample without any pretreatment is 4.2 (log CFU/g) at the 0th day. During storage, the total bacterial counts of samples increased. On the 12th day, the aerobic plate count was: T3 (6.6 log CFU/g) < T2 (7.4 log CFU/g) < T1 (7.8 log CFU/g) < CK (9.1 log CFU/g). The antibacterial effect was: T3 > T2 > T1> CK, and there was a dramatic difference between the T3 treatment group and the CK group (*p* < 0.01). To sum up, blue LED_460nm_ light treatment had an antibacterial effect, and 30 μmol/(m^2^·s) blue LED_460nm_ light treatment had the best antibacterial effect. Furthermore, the antibacterial effect gradually increased with the rise in blue light intensity. Blue light could induce bacterial apoptosis by stimulating endogenous photosensitizers (PSs) and transferring electrons to molecular oxygen to form reactive oxygen species (ROS) [43]. Blue light could also inhibit other rot pathogens, such as *Listeria monocytogenes* [44], *Bacillus subtilis* [45], *Escherichia coli* [46], and so on. As a kind of spoilage bacteria in plants, *pseudomonas* had pectin decomposition activity, which led to freshly cut amaranth spoilage [47]. Hyun et al. [48] found that 460–470 nm LEDs inhibit bacteria mainly by disrupting the cell envelope, causing irreversible damage to bacteria by destroying their ribosomes. As can be seen from Figure 9B, the bacteria in the experimental groups showed a rising trend. The growth rate of *P. fluorescens* in the treated group was lower than that in the control group. The results showed that 30 μmol/(m^2^·s) blue LED_460nm_ light could effectively inhibit the growth of Pseudomonas.

### 3.8. Changes in Microbial Community

**Figure 10:**
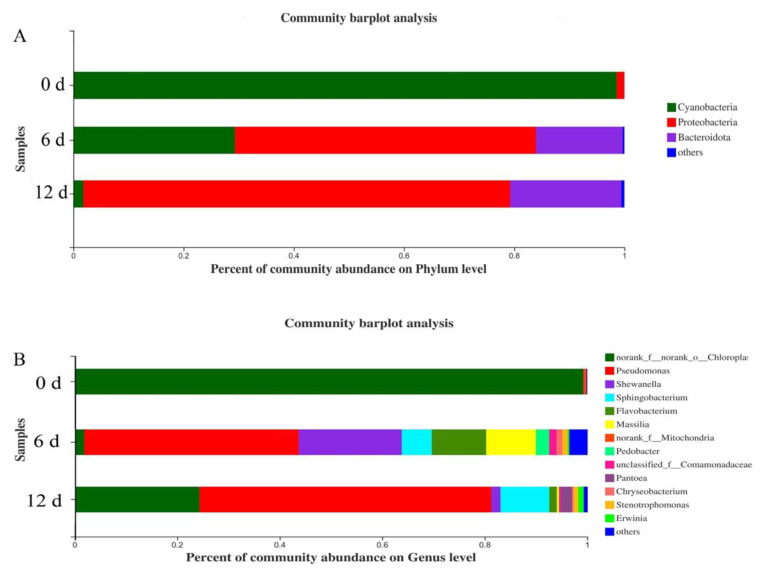
Changes in percent of community abundance on phylum (**A**) and genus (**B**) levels.

RDP classifier and blast analysis were used to classify the OTUs. A total of 43 genera, 32 families, 5 phyla, 6 classes, and 20 orders were identified. Figure 10 showed the relative abundance distribution map of freshly cut amaranth samples under the condition of phyla and genus classification standards. Under the condition of phylum classification standard, the flora of fresh samples mainly include *cyanobacteria*, *Proteobacteria*, and so on. Among them, *Proteobacteria* was the dominant species during the whole storage period, and the relative abundance increased from 0.04% to 77.45%. Under the condition of genus classification standard, the flora of fresh samples mainly includes *Pseudomonas, Shewanella*, *Sphingobacterium*, and so on. During storage, the abundance of *Pseudomonas* increased from 0.04 to 41.83%, and finally to 56.93%, so the dominant spoilage bacteria of freshly cut amaranth was *Pseudomonas*. The relative abundance of other flora decreased gradually. The main reason was that the growth of dominant bacteria was too fast, which inhibited the growth of other microorganisms. *P. fluorescens* had been confirmed to be the dominant spoilage bacteria for many vegetables [49]. The physiological properties of *P. fluorescens* were adapted to the growth environment of vegetables, and its abundance accounted for about 50–90% of spoilage bacteria. In this genus, *P. fluorescens* is the main bacteria causing soft rot and yellowing of freshly cut amaranth [50].

### 3.9. Correlation Analysis

**Figure 11:**
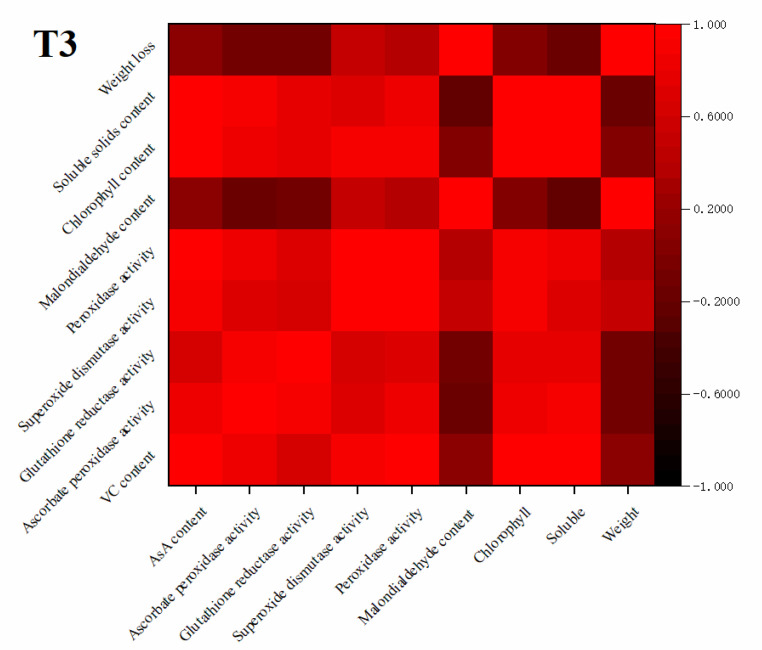
Correlation of AsA content with APX activity, GR activity, SOD activity, POD activity, MDA content, soluble solids content, weight loss, and chlorophll content.

As shown in Figure 11, the AsA content of freshly cut amaranth during storage was significantly and positively correlated with APX activity, SOD activity, and POD activity. In addition, AsA content was positively correlated with physiological indices of soluble solids and chlorophyll content. The results showed that 30 μmol/(m^2^·s) blue LED_460nm_ light treatment of freshly cut amaranth AsA content was closely correlated with AsA–GSH cycle activity, which demonstrated strong oxidative stress properties and enhanced the increase of AsA content. The physiological indicators were also elevated in freshly cut amaranth under blue LED_460nm_ light irradiation to maintain its vital signs. Blue LED_460nm_ light could align the antioxidant capacity of freshly cut amaranth and prolong the shelf life of freshly cut amaranth by increasing the activity of antioxidant enzymes.

## 4. Conclusions

In conclusion, the shelf life of freshly cut amaranth treated with blue LED_460nm_ light irradiation could be effectively prolonged by 2–3 days, and sensory scores were increased to satisfy the consumers. Blue LED_460nm_ light improved the content of chlorophyll and soluble solids, ascorbic acid, and antioxidant capacity. The rise in POD, SOD, APX, and GR activities advanced the antioxidant capacity of freshly cut amaranth. It could effectively inhibit colony reproduction and the growth of dominant spoilage bacteria *Pseudomonas*. The best preservation effect was obtained by 30 μmol/(m^2^·s) blue LED_460nm_ light on freshly cut amaranth compared with the other groups. However, the water loss of freshly cut amaranth irradiated by blue LED_460nm_ was not good enough. In a follow-up study, red light, ultraviolet light, or other different light sources could be used on freshly cut amaranth for further exploration.

## Figures and Tables

**Table 1:** Freshly cut amaranth of each experimental group.

Group	Light Intensity (μmol/(m^2^·s))	LED Band (nm)
CK	-	-
T1	10	460
T2	20	460
T3	30	460

**Table 2:** Sensory evaluation project.

Score	Color	Form	Smell
10	Full and bright color	Crisp	Refreshing fragrance
8	The color is a little dim, but not brown	It’s brittle, but it doesn’t shrink	No fragrance, no peculiar smell
6	Overall acceptable, with occasional browning	Slight atrophy	No fragrance, slightly peculiar smell after careful smelling
4	Browning rate < 1/3	Obvious atrophy, but not serious	Obvious odor, but not serious
2	Browning rate ≥ 1/3	Atrophy serious	Severe odor
0	All browning and the color of mildew spots can be seen	All severely atrophied and moldy	Stench

## Data Availability

We did not report any new data in this review.
